# Effect of Pinocembrin Isolated from Mexican Brown Propolis on Diabetic Nephropathy

**DOI:** 10.3390/molecules23040852

**Published:** 2018-04-09

**Authors:** Jessica Granados-Pineda, Norma Uribe-Uribe, Patricia García-López, María del Pilar Ramos-Godinez, J. Fausto Rivero-Cruz, Jazmin Marlen Pérez-Rojas

**Affiliations:** 1 Facultad de Química, Universidad Nacional Autónoma de México, Ciudad Universitaria, Coyoacán, 04510 Ciudad de Mexico, Mexico; jessygpin@hotmail.com; 2 Instituto Nacional de Ciencias Médicas y Nutrición Salvador Zubirán S.S., 14080 Ciudad de México, Mexico; nofeliauribe@yahoo.com.mx; 3 Subdirección de Investigación Básica, Instituto Nacional de Cancerología, 14080 Ciudad de México, Mexico; pgracia_lopez@yahoo.com.mx; 4 Departamento de Patología Quirúrgica, Instituto Nacional de Cancerología, 14080 Ciudad de México, Mexico; pilyrg@gmail.com

**Keywords:** pinocembrin, propolis, diabetic nephropathy, renal failure, diabetes

## Abstract

Propolis is a resinous beehive product that has been used worldwide in traditional medicine to prevent and treat colds, wounds, rheumatism, heart disease and diabetes. Diabetic nephropathy is the final stage of renal complications caused by diabetes and for its treatment there are few alternatives. The present study aimed to determine the chemical composition of three propolis samples collected in Chihuahua, Durango and Zacatecas and to evaluate the effect of pinocembrin in a model of diabetic nephropathy in vivo. Previous research demonstrated that propolis of Chihuahua possesses hypoglycemic and antioxidant activities. Two different schemes were assessed, preventive (before renal damage) and corrective (once renal damage is established). In the preventive scheme, pinocembrin treatment avoids death of the rats, improves lipid profile, glomerular filtration rate, urinary protein, avoid increases in urinary biomarkers, oxidative stress and glomerular basement membrane thickness. Whereas, in the corrective scheme, pinocembrin only improves lipid profile without showing improvement in any other parameters, even pinocembrin exacerbated the damage. In conclusion, pinocembrin ameliorates diabetic nephropathy when there is no kidney damage but when it is already present, pinocembrin accelerates kidney damage.

## 1. Introduction

Around the world there are 425 million people that have diabetes mellitus [1]. According to Chronic Disease Survey of 2016, there was a 9.4% prevalence rate of diabetes in Mexicans from 20–65 years of age [2]. Diabetes is second cause of death in Mexico and has shown an increasing tendency over the past half century [3].

Diabetic nephropathy (DN) is the most important vascular long-term complication of diabetes and it is the leading cause of end stage renal disease in the western societies [4]. In Mexico, retinopathy is the most frequent complication, nevertheless DN represent the major expenditure in health care systems because of the costs of peritoneal dialysis and hemodialysis [5]. The main features of DN are persistent urinary protein, hypertension and progressive renal function loss [6].

The main treatment for DN is glycemic, hypertension and dyslipidemia control and/or the use of renin-angiotensin-aldosterone blockers, mainly for the prevention of microalbuminuria and reduction of cardiovascular mortality [7]. Unfortunately, the drugs cause undesirable side effects and the patients still reach end stage renal disease [8]. For this reason, it is necessary to find alternatives to prevent the early appearance of DN and to stop its progression.

Traditional Medicine continues to be the most used alternative by the population, such as propolis. Propolis (bee glue), a sticky dark-colored hive product collected by bees from living plant sources, is well known to possess pharmacological activities such as antibacterial, antifungal, antioxidant, antitumoral and anti-inflammatory [9]. It has been used in folk medicine as early as 300 B. C. Egyptians used it as an ingredient in the formula employed to embalm cadavers. Greek, Roman and Arab physicians also used it as an antiseptic and to treat wounds. Incas employed it as anti-pyretic agent and in seventeen century this natural product was listed as an official drug in London pharmacopoeias [9,10,11]. In recent years, it has gained popularity as a health drink and is used extensively in food and beverages in various parts of the world where is claimed to improve human health and to prevent diseases such as diabetes and cancer [12].

To date, at least 300 compounds have been identified in different propolis samples [11]. This complex mixture contains a variety of chemical compounds such as flavonoid aglycones, phenolic acids and their esters, phenolic aldehydes, alcohols, ketones, sesquiterpenes, coumarins, steroids, amino acids and inorganic compounds [13]. The results revealed that the propolis composition depends on the geographical variations and is strongly related with the flora surrounding the hive [11]. In general, propolis composition is related to that of the bud exudates collected by honeybees from poplar, birch, beech, horse chestnut, alder and conifer trees [14]. The main constituents of propolis in North America are flavonoids and phenolic acid esters [15]. 

In Mexico propolis is prepared in several forms, including syrups, tinctures and creams as alternative to improve health and prevent diseases, however data about the chemical composition and biological activity of Mexican propolis are limited [15,16,17,18,19,20]. There are reports of the chemical composition of propolis from Sonora [15,16], Yucatán [17,19], Quintana Roo [18,19], Chiapas [19] and Chihuahua [20], in which flavonoids, terpenoids, phenolic acids and their esters have been isolated and identified. The reported biological activity for Mexican propolis is antibacterial [15,18], anticancer [16], antifungal [18], antioxidant [18,19], anti-inflammatory [19] and hypoglycemic [20]. Nevertheless, Mexico has a wide range of weather and flora, which have an impact on propolis composition.

In the last decade, it has been proposed that propolis has an antihyperglycemic effect and might prevent biochemical and structural alterations in animal models and in diabetic patients [21,22,23,24]. The main reported flavonoids in temperate propolis are pinocembrin, galangin and chrysin [11]. The flavonoids quercetin and chrysin have been demonstrated to diminish the severity of renal injury by decreasing the expression of inflammatory cytokines involved in the progression of DN [8,25]. Pinocembrin is found in plants and in propolis, where is considered the marker compound of poplar type propolis [11]. It has neuroprotective, anti-inflammatory, hepatoprotective, antihyperlipidemic and vasorelaxant properties [26].

To our knowledge, this is the first study on the chemical composition of propolis collected in the North of Mexico and on the effect of pinocembrin on renal function in streptozotocin-induced diabetic rats.

## 2. Results

### 2.1. Isolation of Flavonoids from EEP Samples of Propolis

The studied propolis samples were collected in the North of Mexico (Durango, Chihuahua, Zacatecas).

Durango’s EEP yielded pinocembrin (**1**), pinobanksin (**2**) and chrysin (**3**); Zacatecas’ EEP gave pinocembrin (**1**), isorhamnetin (**4**), pinobanksin-5-methylether (**5**) alpinetin (**6**). Chihuahua’s EEP yielded pinocembrin (**1**), alpinone (**7**), pinostrobin (**8**), galangin-5-methylether (**9**) and kaempferide (**10**).

### 2.2. Total Phenolics and Total Flavonoids of the EEP Samples

The content of total phenolics in EEP samples ranged between 109 ± 2 and 139 ± 1 mg eq. GA/g extract. While the content of total flavonoids was between 70 ± 1 and 90 ± 2 mg eq. Q/g extract (Table 1).

### 2.3. Antioxidant Capacity of the EEP Samples

The antioxidant capacity of the extracts is expressed in reference to the antioxidant Trolox. The values obtained for the samples, in the DPPH bleaching assay, ranged between 975 ± 33 and 1145 ± 10 TE/g extract. The total antioxidant given by the FRAP assay ranged between 13 ± 1 and 21 ± 1 TE/g extract. For the β-carotene bleaching assay the values obtained were between 39.1 ± 7.7 and 45.9 ± 12.5% (Table 1).

Total phenolics content expressed as milligrams of equivalents of gallic acid per gram of extract (mg eq. GA/g extract). Total flavonoid content expressed as milligrams of equivalents of quercetin per gram of extract (mg eq. Q/g extract). The antiradical efficiency was calculated with DPPH bleaching assay and expressed as Trolox equivalents per gram of extract (TE/g extract). The total antioxidant capacity in the FRAP assay is given as Trolox equivalents per gram of extract (TE/g extract). The percentage of Antioxidant Activity (%AA) was measured with the β-carotene bleaching assay. Values are expressed as means ± standard error, *n* = 3.

### 2.4. Effect of Pinocembrin on Body Weight, Survival and Blood Glucose

**Figure 1:**
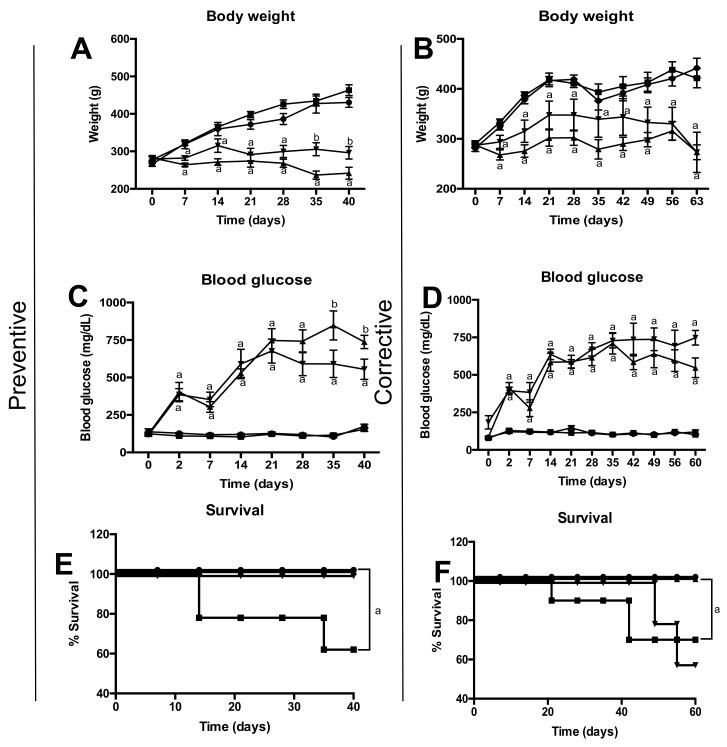
Metabolic parameters in preventive scheme and corrective scheme. (**A**) Weekly body weight monitoring in the preventive scheme; (**B**) Weekly body weight monitoring in the corrective scheme. (**C**) Weekly blood glucose monitoring in the preventive scheme; (**D**) Weekly blood glucose monitoring in the corrective scheme; (**E**) Overall survival throughout preventive scheme; (**F**) Overall survival throughout corrective scheme. (●) Control, (■) Pinocembrin, (▲) Diabetic, (▼) Diabetic + Pinocembrin, ^a^ *p* < 0.05 vs. Control, ^b^ *p* < 0.05 vs. Diabetic, *n* = 6–10.

Preventive scheme represents a beginning and/or maintenance of diabetes before developing diabetic nephropathy. Figure 1A,C show body weight and glucose level in the four groups of study. Diabetic group shows significant decrease in body weight in compared to the control group. Pinocembrin treatment did not show significant effect on these parameters, despite observing slight improvements; pinocembrin partially avoided the increases of glucose levels in compared to the diabetic group. These effects are reflected in an increase of percentage of survival (Figure 1E). Corrective scheme depicts an uncontrolled diabetes or/and advanced stage of the disease, where there is already damage in renal structure. In this treatment of scheme, pinocembrin did not have effect on body weight neither in blood glucose level (Figure 1B,D). Moreover, the glycaemia increased more in the rats, which was reflected in a decrease in their survival (Figure 1F).

### 2.5. Effect of Pinocembrin on the Liver

**Figure 2:**
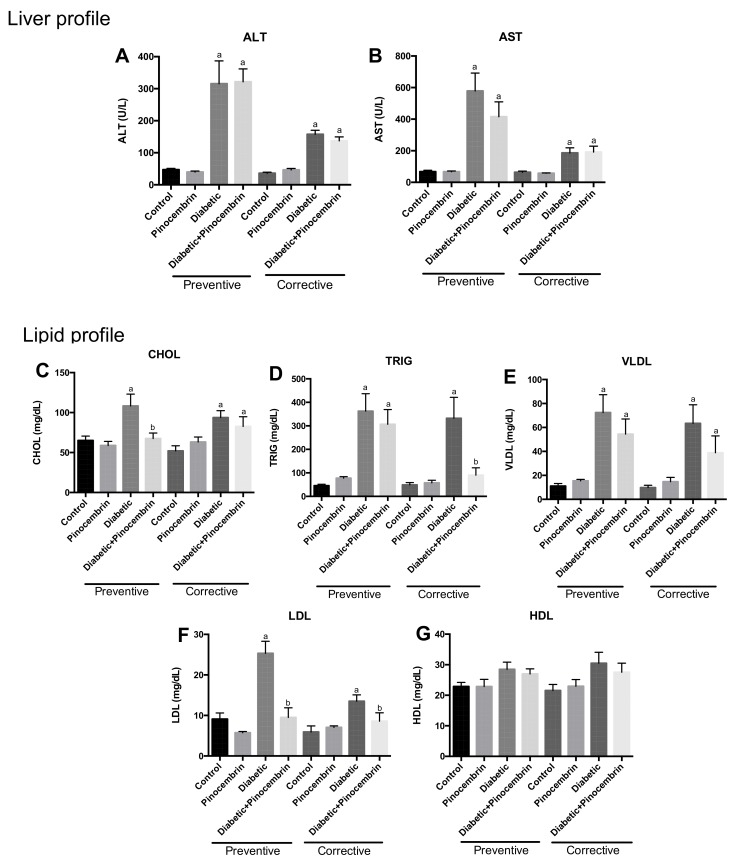
Hepatic profile in the schemes of treatment with pinocembrin, 40th day in the preventive scheme and 60th day in the corrective scheme. (**A**) Levels of ALT in preventive and corrective scheme (**B**) Levels of AST in preventive and corrective schemes. Lipid profile in the schemes of treatment with pinocembrin, 40th day in the preventive scheme and 60th day in the corrective scheme; (**C**) Cholesterol (CHOL) levels in preventive and corrective schemes; (**D**) Triglycerides (TRIG) levels in preventive and corrective schemes (**E**) Very Low Density Lipoprotein (VLDL) levels in preventive and corrective schemes (**F**) Low Density Lipoprotein (LDL) levels in preventive and corrective schemes (**G**) High Density Lipoprotein (HDL) levels in preventive and corrective schemes. ^a^ *p* < 0.02 vs. Control; ^b^ *p* < 0.01 vs. Diabetic, *n* = 6–9.

Figure 2A,B show the values of hepatic enzymes measured in both schemes. AST and ALT were quantified in serum of all groups. In both schemes, the rats administrated with streptozotocin showed an increase of these parameters in comparison with the control group. Pinocembrin could not avoid this increase.

### 2.6. Effect of Pinocembrin on Lipids

Figure 2C–E depict the values of lipids in preventive and corrective schemes. Cholesterol, triglycerides, VLDL and LDL concentrations were significantly greater in the diabetic group in compared with the control group in both schemes; but there was no significant change in HDL. Pinocembrin administered in control group did not affect any lipid values. Administration of this compound to diabetic rats avoided the increase in most of the parameters studied in the preventive scheme. While in the corrective scheme, pinocembrin showed a significant reduction of triglycerides and LDL levels in compared to the diabetic group (Figure 2D,F).

### 2.7. Effect of Pinocembrin on Renal Function

**Figure 3:**
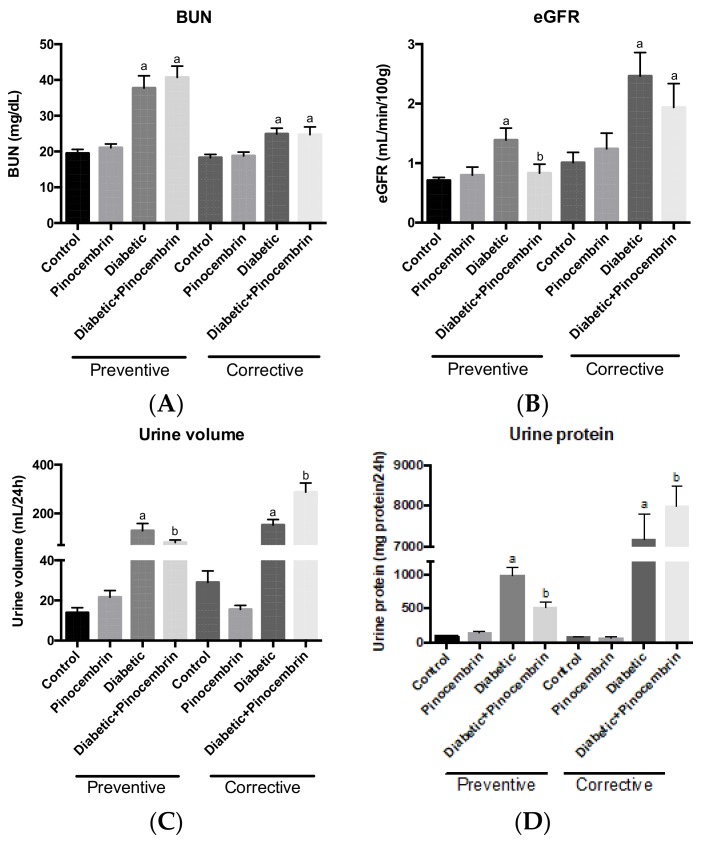
Kidney profile in the schemes of treatment with pinocembrin, 40th day in the preventive scheme and 60th day in the corrective scheme. (**A**) Blood Urea Nitrogen (BUN) levels in the preventive and the corrective schemes; (**B**) Estimated Glomerular Filtration Rate (eGFR) in the preventive and the corrective schemes; (**C**) Urine volume measured in the preventive and the corrective schemes. (**D**) Urine protein levels in the preventive and the corrective schemes. ^a^ *p* < 0.02 vs. Control, ^b^ *p* < 0.002 vs. Diabetic, *n* = 6–9.

Figure 3A–D show the parameters of renal function in the preventive and corrective scheme, respectively. In the preventive scheme, BUN and eGFR were significantly elevated in diabetic animals compared to the control group. This is an indication of hyperfiltration, an early sign of renal injury. Pinocembrin administrated to diabetic animals was able to maintain normal eGFR, meaning that pinocembrin avoided hyperfiltration. Both urinary volume and urinary protein were significantly higher in diabetic group in compared to the control group; administration of pinocembrin to diabetic rats partially avoids that increase.

In the corrective scheme, a rise in BUN was observed in diabetic rats as compared to control rats. Pinocembrin could not avoid the increase in BUN in diabetic animals. There were no significant differences in eGFR among the groups. Urinary volume and urine protein were significantly greater in the diabetic group in compared to the control group (Figure 3A–D). Pinocembrin administrated to diabetic animals seems to heighten both parameters, which suggests that pinocembrin augments renal injury.

### 2.8. Histopathological Analysis

**Figure 4:**
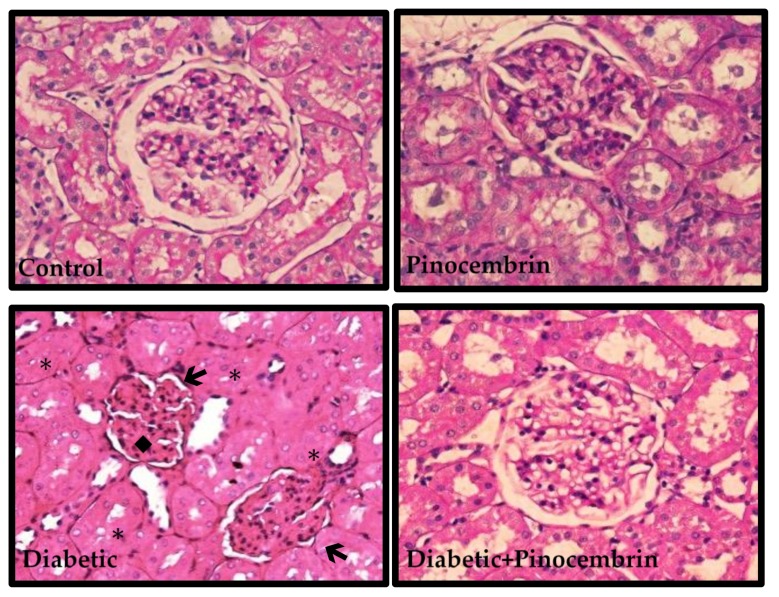
Representative photomicrographs of glomerulus structure in the preventive scheme. Renal structure stained with PAS. Symbols (←) Marked lobulation of the glomerulus, (♦) extracellular matrix accumulation, (*) occluded tubules.

In the preventive scheme, observation of PAS stain showed no histological alterations in the kidneys of control and pinocembrin group. In diabetic rats, there is a marked lobulation of the glomeruli, mesangial expansion, occlusion and vacuolization of tubules, as shown in Figure 4. In diabetic animals treated with pinocembrin, histological injury was prevented and renal morphology was similar to that in control group.

**Figure 5:**
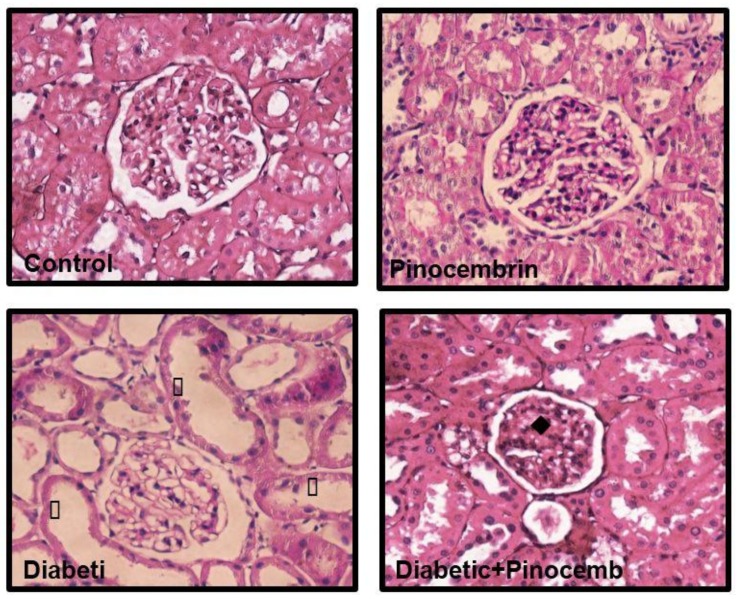
Representative photomicrographs of glomerulus structure in the corrective scheme. Renal structure stained with PAS. Symbols (♦) extracellular matrix accumulation, (▯) loss of brush border.

In the corrective scheme, control and pinocembrin group showed normal renal structure (Figure 5). While in diabetic group, there was vacuolization in tubules cytoplasm, mesangial expansion and fibrosis. In diabetic rats treated with pinocembrin the damage was not prevented.

**Figure 6:**
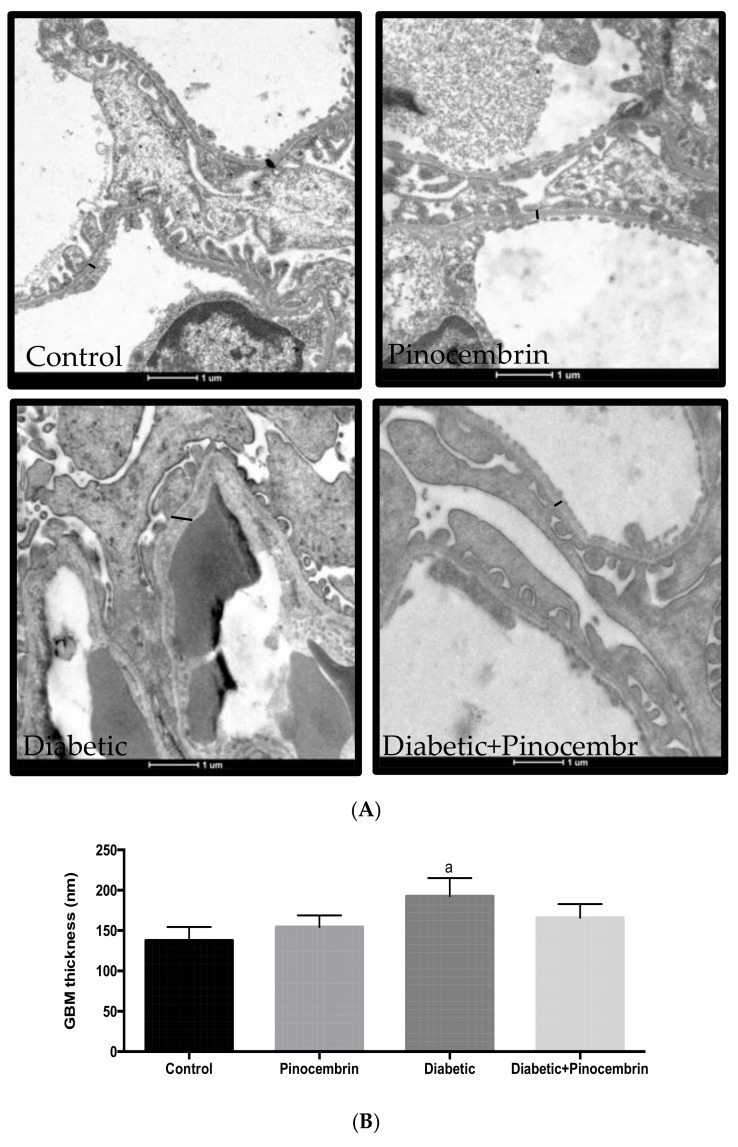
Representative photomicrographs of glomerulus ultrastructure in the preventive scheme. (**A**) Representative images of electron microscopy of preventive model, where measurements of GBM are expressed in nm. Scale bar = 1 μm; (**B**) Means ± SEM. ^a^ *p* < 0.01 vs. Control, *n* = 3–5.

Figure 6A shows the representative micrographs under electron microscopy of experimental groups. Control group exhibits the filtration barriers. Diabetic group show segmental foot process fusion and significant glomerular basement membrane thickening in compared to the control group (Figure 6B).

### 2.9. Biomarkers of Renal Damage

**Figure 7:**
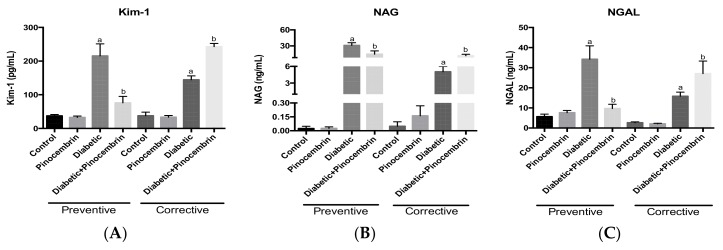
Biomarkers of renal damage in the schemes of treatment with pinocembrin, 40th day in the preventive scheme and 60th day in the corrective scheme. (**A**) Kidney injury molecule-1 (Kim-1) levels in the preventive and the corrective schemes; (**B**) *N*-acetyl-β-glucosaminidase (NAG) levels in the preventive and the corrective schemes; (**C**) Neutrophil gelatinase-associated lipocalin (NGAL) levels in the preventive and the corrective schemes. ^a^ *p* < 0.02 vs. Control, ^b^ *p* < 0.005 vs. Diabetic, *n* = 5.

Figure 7 depicts the values of biomarkers of renal damage in urine within preventive scheme. There was no significant difference between control group and pinocembrin group. Diabetic group has significant increases in the levels of Kim-1, NAG and NGAL in compared to the control group. Whereas, pinocembrin avoids the elevation of all the biomarkers.

Figure 7 shows the values of biomarkers of renal damage in urine within the corrective scheme. As in the preventive scheme, in this scheme there was no significant difference between control and pinocembrin group; whereas the diabetic group has significant increases in the levels of the biomarkers. While pinocembrin does not diminish any of the biomarkers, conversely it increases them even more; this indicates that pinocembrin increases renal damage instead of avoiding it.

### 2.10. Oxidative Stress

Since pinocembrin is an antioxidant compound, oxidative stress was evaluated in both schemes. In Table 2 are shown the oxidative stress parameters for preventive scheme: urinary hydrogen peroxide excretion, MDA content in plasma and kidney were measured. We found that the administration of pinocembrin did not modify any of the parameters studied. While on the contrary in the untreated diabetic group there was a significant increase of all of them in compared to the control group. Pinocembrin lead to a significant diminution in MDA content in plasma and kidney, without showing changes in urinary hydrogen peroxide excretion.

Instead in the corrective scheme (Table 3), like the other parameters, pinocembrin administration exacerbates the damage.

## 3. Discussion

In the latest years there has been a dramatic increase in obese population, which in turn has produced an increase in diabetic population in earlier ages [1]. These patients develop macrovascular and microvascular complications; among the later complications DN has greater impact on the quality of life and economic cost in patients. In consequence, population looks up to traditional medicine and herbal remedies to attend health care. The World Health Organization estimates that in industrialized regions over 50% of the population have used complementary or alternative medicine at least once. While countries like Africa and Latin America use traditional medicine to meet their primary health needs [27]. Propolis has gained popularity in recent years because of its wide range of biological and pharmacological activities such as antioxidant, anti-inflammatory and antibacterial agent [28]. 

The composition of propolis is highly variable but unsubstituted-B ring flavonoids, such as pinocembrin, are characteristic of poplar type propolis from temperate regions [11].

There are only five reports of chemical composition of Mexican propolis. Li and coworkers [16] isolated pinocembrin, pinobanksin 3-acetate, tectochrysin, galangin and chrysin, aromatic acids and their esters from propolis collected in Sonora. Lotti and coworkers [17] isolated pinocembrin along with isoflavans and pterocarpans from propolis collected in Yucatán. Boisard and coworkers [18] studied a sample from Quintana Roo and they identified by HPLC-DAD pentacyclic triterpenoids such as α-amyrenone I, α-amyrin IV, fucosterol and β-sitosterol. Guzmán-Gutiérrez and coworkers [19] isolated epoxypinocembrin, pinostrobin, izalpinin, pinocembrin, kaempferol, rhamnetin and aromatic acids from Chiapas and Yucatán. Rivera-Yáñez and coworkers [20] analyzed a sample from Chihuahua by HPLC-DAD—they identified naringin, narigenin, kaempferol, quercetin, acacetin, luteolin, chrysin and pinocembrin. In contrast to Rivera-Yáñez and coworkers [20], we found only pinocembrin, this probably occurred because of the site of collection and the different weathers present along Chihuahua province. The samples from Quintana Roo and Yucatán may be considered apart from the other provinces of Mexico since the botanical sources are not poplar trees but *Busera simaruba*, *Lysiloma latisiliquum* and *Dalbergia sp* [17,18]. Pinocembrin (**1**) appears to be the common compound among the reports of Mexican propolis. The isolation process of the three samples collected in different provinces of the North of Mexico yielded mainly flavonoids. We found again that pinocembrin (**1**) was the common compound in the three samples and moreover, it was obtained in the highest yield (Chihuahua, 1.76 g, Durango, 2.44 g and Zacatecas, 1.53 g). Other nine flavonoids (**2**–**10**) were isolated from the samples studied. These compounds were identified as pinobanksin (**2**) and chrysin (**3**) isorhamnetin (**4**), pinobanksin-5-methylether (**5**) alpinetin (**6**), alpinone (**7**), pinostrobin (**8**), galangin-5-methylether (**9**) and kaempferide (**10**). All these isolated compounds **1**–**10** (Figure S2) were identified through a comparison with the NMR data in the literature (Figure S1, Table S1). The common compound among the propolis samples was pinocembrin (**1**) and it was obtained in the highest yield of all the compounds isolated.

The total phenolic content is used in routine screening of natural products and measures the sample’s reducing capacity [29]. The samples tested range between 109 and 139 mg eq. GA/g extract, in contrast to the 314 mg eq. GA/g extract found by Rivera-Yáñez and coworkers [20]. Among the phenolics, flavonoids are suggested to be responsible for biological activities, therefore we assessed the flavonoid content of the samples. The samples ranged between 70 ± 1 and 90 ± 2 mg eq. Q/g extract. These results are higher compared with the report of Rivera-Yáñez and coworkers [20] (6.25 mg eq. Q/g extract). In addition, the content of total phenolics and total flavonoids of the three samples meet the requirements of the Mexican legislation NOM-003-SAG/GAN-2017 “Propóleos, producción y especificaciones para su procesamiento” [30].

The total phenolic and flavonoid content correlate with the antioxidant capacity assay [31]. When assessing in vitro antioxidant activity, it is recommended to use more than one assay and to include single electron transfer (SET) and hydrogen atom transfer (HAT)- based mechanisms [32]. We performed the DPPH bleaching assay, FRAP assay and β –carotene bleaching assay. The antioxidant capacity found in the EEP samples of Chihuahua, Durango and Zacatecas is in agreement with previous reports for temperate poplar propolis Croatian propolis [23], French propolis [28] and Argentinean propolis [33].

Propolis is used as a whole in Traditional Medicine, however its composition greatly varies with the site of collection as we demonstrated with our study of three samples of the same type of propolis (temperate propolis) and the same country. For that reason, we proposed to study the effects of a single compound that is present in all samples. In the present study, pinocembrin was isolated from Mexican propolis collected in three different locations and evaluated in a model of DN in rats. There are two approaches to combat DN, the first is to try to avoid or delay its early appearance and the second is to reverse and/or slow down its progression once it has been diagnosed. For that reason, in this study we explored both stages using the most abundant compound of propolis; in order to determine if this compound is responsible for the beneficial effects reported.

We demonstrated that pinocembrin shows, in a preventive scheme, little effect upon body weight and blood glucose (Figure 1A) and avoids death (Figure 1E). These results are the opposite of those reported for the ethanolic extract of propolis (EEP). EEPs increase body weight nearly to normal values and reduce blood glucose, in a scheme similar to our preventive scheme [21,23]. Chrysin, another flavonoid isolated from propolis, does not lower blood glucose but stops weight loss [8]. This data suggest that is the mixture of all compounds of propolis, which make up the anti-hyperglycemic effect and in turn prevent body weight loss.

Diabetes is a metabolic alteration of carbohydrates, lipids and proteins; therefore, diabetic nephropathy will reflect all these biochemical alterations and mainly those related to renal tissue. In this study, we measured renal, lipid and hepatic profiles. We found that pinocembrin ameliorated renal function (Figure 3B–D). This result is in accordance with previously reports of flavonoids ameliorate renal function in DN [8,25].

The overload of plasmatic proteins in renal tubular cells causes the production of pro-inflammatory and pro-fibrotic mediators leading to renal damage [34]. Thus, the levels of renal biomarkers: Kim-1, NAG and NGAL, were quantified as indicative of tubular damage. It has been already reported that Kim-1, NAG and NGAL are elevated in diabetic patients [35,36]. Herein, diabetic group increases all of them and pinocembrin avoid the rise (Figure 7). So, pinocembrin could be proposed as a renoprotector agent.

Since albuminuria is correlated with the structural changes in the glomerulus and pinocembrin reduced the observed albuminuria, there must have been amelioration in the glomerular filtration barrier. So, we assessed the GBM, which is the first measurable change at the early onset of diabetes [6]. The treatment with pinocembrin leads to the reduction of GBM thickness, although it did not reach statistical significance (Figure 6B).

The onset of renal disease is multifactorial; it involves several factors as hypertension, oxidative stress and hyperlipidemia. Dyslipidemia was observed in the diabetic group and pinocembrin normalized the levels (Figure 2C–G). Those results correlate with a report in which the reduction in hypertriglyceridemia in obese Zucker rats was related to the reduction of glomerular injury [37]. These results suggest that the hypolipidemic effect of pinocembrin contributed to stopping renal damage in the preventive scheme.

Hepatic damage was found in animal models of STZ and in DM1 patients [38]. Pinocembrin did not show effect on the liver enzymes studied (Figure 2A,B). These result contrast with the findings in propolis, where administration of EEP avoided the rise of transaminases [22]. It also differs from the results found by Rauter [38], in which the flavonoids tested lowered the levels of those enzymes in hyperglycemic rats. So, these results anew support our findings that it is the mixture of compounds of propolis, which have the effect.

Hyperglycemia is responsible for the production of oxidative stress by multiple pathways. Oxidative stress attacks lipid membranes leading to lipid peroxidation and generating reactive products that have been implicated in diabetic complications [39]. MDA is a final product of lipid peroxidation widely employed as oxidative stress marker. In this study, pinocembrin significantly reduced MDA in both plasma and kidney tissue (Table 2). This agrees with previous reports in which pinocembrin inhibited the formation of thiobarbituric acid reactive substances in isolated mitochondria and in the hippocampus of ischemia/reperfused rats [40]. Additionally, pinocembrin exerts its antioxidant activity by capturing reactive oxygen species (ROS) and restoring glutathione levels [41]. H_2_O_2_ is a ROS that has been proposed as a biomarker of global oxidative stress given that MDA is only a marker of lipid oxidative stress. In this study, pinocembrin did not attenuate its rise (Table 2). In DN, Nicotinamide Adenine Dinucleotide Phosphate oxidase 4 (Nox4) greatly contributes to superoxide production in the renal cortex and is later dismutated to O_2_^−^ [42]. There are no reports of pinocembrin superoxide scavenging activity. Our results show that pinocembrin might not scavenge the superoxide produced, which in turn gives high levels of H_2_O_2_.

In summary, in the preventive scheme pinocembrin was able to improve survival, partially decrease blood glucose, lipids as well as renal function and renal structure before renal damage. A possible mechanism of protection is through diminution of oxidative stress, which is known to be a main cause of initiation and progression of renal injury.

Once we showed that pinocembrin ameliorates renal damage in preventive scheme, we proposed to assess whether pinocembrin can reverse and/or stop the damage once the structural damage is established. 

Pinocembrin given to diabetic rats reduced triglycerides and LDL nearly back to normal levels (Figure 2D,F). Nevertheless, there were not improvements in renal parameters, even it seems that pinocembrin worsens damage because of the significant higher levels of urinary protein, urinary volume, Kim-1, NAG and NGAL in compare to untreated diabetic rats. The harmful observed effect of pinocembrin in this model could be explained by the prooxidant effect of antioxidants, which happens under certain circumstances. The possible mechanisms of the prooxidant effects of flavonoids are the enhancement of the Fenton reaction, the inhibition of mitochondrial respiration, autoxidation and the oxidation of low molecular antioxidants [43]. The increased levels of urine H_2_O_2_ excretion indicate that pinocembrin increases oxidative stress and consequently exacerbates the damage. 

In the literature, there are no reports of propolis or pure flavonoids administrated after structural damage. Hence this is the first report aimed to investigate late alterations in STZ induced DN. We found that pinocembrin aggravates the damage; produced for this reason it must not be given to patients in the late stage of this illness.

Further investigations relating to in vivo activity, toxicity and chemical composition of EEP of Chihuahua, Durango and Zacatecas are currently underway.

## 4. Materials and Methods 

### 4.1. Chemicals and Reagents

Streptozotocin, bovine serum albumin, bicinchoninic acid, CuSO_4_, trimethoxypropane, methanesulfonic acid, HCl, FeCl_3_, 1-methyl-2-phenyl indole, acetonitrile, methanol, acetone, dichloromethane were obtained from Sigma Aldrich Co. (St. Louis, MO, USA). All other reagents were obtained from commercial sources.

### 4.2. Propolis Samples

Propolis produced by *Apis mellifera* was collected in experimental apiaries located in Gomez-Palacio, Durango in November 2014; Fresnillo, Zacatecas in November 2013 and Parral, Chihuahua in November 2015; Mexico.

### 4.3. Extraction and Isolation 

The samples were extracted independently by maceration with ethanol 96%, filtered and concentrated under vacuum. Each ethanolic extract of propolis (EEP) (Durango, 43.8 g; Zacatecas, 80.0 g and Chihuahua, 52.1 g) was individually subjected to vacuum liquid chromatography (VLC) over 350 g of silica gel (Merck) and eluted with a gradient mixture of dichloromethane–acetone (1:0 → 0:1). The particular isolation procedure for each EEP is described as follows.

EEP of Durango. The VLC gave 30 fractions that were gathered according to its TLC similarity into ten combined fractions (I-X). Fraction II and III eluted with 95–5% dichloromethane-acetone, from which a white solid spontaneously precipitated, then it was recrystallized from dichloromethane to give **1** (2.44 g). The mother liquor was subjected to VLC over 350 g of silica gel and eluted with a gradient mixture of dichloromethane–acetone (1:0→0:1) to obtain 23 fractions that were gathered according to its TLC similarity into ten pooled fractions (FIII-1–FIII-10). Fraction FIII-6 was rechromatographed over polyamide **6** and eluted with a gradient of ethanol–water (1:0→0.5:0.5) to give twenty fractions (FIII-6-1–FIII-6-20), fraction FIII-6-2 afforded 2 (6.0 mg) and FIII-6-10 3 (30.0 mg).

EEP of Zacatecas. The EEP was subjected to a VLC column chromatography gave 52 fractions that were gathered according to its TLC similarity into eight combined fractions (FI-FVIII). From fraction IV, which was eluted with dichlomethane–acetone 95:5, a white solid spontaneously precipitated; then it was recrystallized from dichloromethane to give **1** (1.53 g). From fraction VI a white powder precipitated (30.0 mg), it was filtered and washed with methanol to give a mixture, that was separated by a polyamide 6 column eluted with ethanol that gave **5** (5.0 mg) and **6** (10.0 mg). The mother liquor was rechromatographed on Sephadex LH-20 with methanol to give five pooled fractions (FVI-1–FVI-5), fraction FVI-5 gave **4** (50.0 mg).

EEP of Chihuahua. The VLC gave 19 fractions that were gathered according to its TLC similarity into six combined fractions (FI-FVI). A white solid spontaneously precipitated from Fraction IV. The solid was filtered and recrystallized from dichloromethane to give 1 (1.51 g). Mother liquor of Fraction IV was rechromatographed on Sephadex LH-20 and eluted with methanol to give 32 fractions that were gathered according to its TLC similarity into eight pooled fractions FIV-1–FIV-8. Fraction FIV-3 was subjected to column chromatography over polyamide 6 and eluted with ethanol, fraction FIV-3-1 gave 7 (190.4 mg), FIV-3-3 8 (47.6), fraction IV-3-7 gave 9 (9.2 mg) and FIV-3-8 gave 10 (10.0 mg).

### 4.4. Total Phenolic Content

The total phenolic content of propolis was determined as described by Singleton and Rossi [44]. Briefly, 20 μL of the extract (1 mg/mL) and 100 μL of Folin-Ciolcateau reagent 0.2 N were mixed well for 5 min and 80 μL of 7.5% sodium carbonate solution was added. The plate was covered and incubated in the dark (at room temperature) during 30 min. The absorbance was measured at 760 nm with a spectrophotometric microplate reader. Distilled water was used as a blank. All the determinations were performed in triplicates. The obtained absorbances were interpolated in a calibration curve (*y* = 0.0036 + 0.0331, R^2^ = 0.9972) of gallic acid. The results were expressed as mg equivalents of gallic acid/g of dry extract of propolis (EEP).

### 4.5. Total Flavonoid Content

The concentration of flavonoids was achieved using the method described by Marquele et al. [45] using the aluminum chloride reagent. A volume of 100 μL of extract was mixed with 100 μL of aluminum chloride solution (2% in methanol). After incubation for 30 min at room temperature, the absorbance was read at 415 nm and concentrations of flavonoids were determined from a calibration curve obtained with quercetin. The obtained absorbances were interpolated in a calibration curve (*y* = 0.017 + 0.0293, R^2^ = 0.9985) of quercetin. The results were expressed as mg equivalents of quercetin/g of dry extract of propolis (EEP). 

### 4.6. Antioxidant Capacity

#### 4.6.1. DPPH Bleaching Assay

The antioxidant DPPH radical scavenging activity was investigated according to the method described in the literature [46]. Briefly, an ethanolic solution of 0.208 mM was mixed with 0.1 mL of different concentrations of extracts or pure compounds. The 96 well plate was incubated in the dark at room temperature for 20 min and the absorbance was recorded at 540 nm. The percentage inhibition of the DPPH by each sample was calculated considering the percentage of steady DPPH in solution after the reaction [% inhibition = 100 (A_control_ − A_sample_)/A_control_]. All the determinations were performed in triplicates. The IC_50_ values were calculated from the relationship curve of scavenging activities (%) versus concentrations of respective sample curve. 

#### 4.6.2. FRAP Assay

The FRAP assay was performed according to literature [47]. Briefly, the working solution was prepared by mixing acetate buffer 300 Mm pH 3.6, 2,4,6-tris(2-pyridil)-s-triazine 10 mM and aqueous solution of FeCl_3_ 20 mM, in a proportion of 10:1: respectively. 180 μL of the working solution and 20 μL of the EEP (0.5 mg/mL) were mixed and incubated for 30 min in the dark. The reading was made at 595 nm.

#### 4.6.3. β-Carotene Bleaching Assay

The assay was performed according to the literature [48]. Briefly, a β-carotene solution (10 mg/mL, CHCl_3_) was added to a boiling flask with 20 mg of linoleic acid and 200 mg of Tween 40. The CHCl_3_ was removed under vacuum and the residue was mixed with 50 mL distilled water to obtain an emulsion. The EEP samples (10 mg/mL, 0.1 mL) were added to a tube with the emulsion and the absorbance of the mixture was immediately recorded at 470 nm. The mixture was subjected to a water bath at 50 °C for 1 h and the absorbance was recorded again. The control consisted on 0.1 mL of distilled water. The antioxidant activity (%AA) was calculated %AA = 100 (DR_c_ − DR_s_)/DR_c_, where DR_c_ is the degradation rate of the control [ln(a/b)/60], DR_s_ is the degradation rate of the sample, *a* is the absorbance at t = 0 and *b* is the absorbance at t = 1 h.

### 4.7. In Vivo Experiments

#### 4.7.1. Animals

All procedures of animal handling were conducted in accordance to the Official Mexican Guide for Animal Experimentation and Care (NOM-062-ZOO-1999) and the Guide for the Care and Use of Laboratory Animals (National Research Council (US) Committee for the Update of the Guide for the Care and Use of Laboratory Animals, 2011) [49]. Biological hazardous residues were discarded according to the corresponding guide (NOM-087-ECOL-SSA1-2001). Male Wistar rats (250–280 g of body weight) were purchased from Instituto Nacional de Ciencias Médicas y Nutrición “Salvador Zubirán.” The animals were housed at 25 °C, 70% humidity and a 12 h light–12 h dark cycle and they had free access to water. They were fed with standard laboratory chow (Harlan, Hong Kong, China).

#### 4.7.2. Experimental Design

Rats were administrated with streptozotocin (STZ) in citrate buffer (60 mg/kg, single dose, i.p.) to produce diabetes. Forty-eight hours after STZ injection, blood glucose was measured with a commercial glucometer (ACCU-CHEK, Switzerland) after twelve hours of fasting. The rats with a glycaemia ≥ 126 mg/dL (NOM-015-SSA2-2010) were randomly divided into two groups: Diabetic group and Diabetic + Pinocembrin group. Two schemes were assessed; preventive and corrective scheme and the groups were formed as follows:

I. Preventive scheme: (a) Control (*n* = 7), orally administrated with carboxymethylcellulose (CMC) 0.5%; (b) Pinocembrin (*n* = 7), treated orally with pinocembrin 10 mg/kg (suspended in CMC 0.5%); (c) Diabetic group (*n* = 8), orally administrated with CMC 0.5%; (d) Diabetic + Pinocembrin group (*n* = 8) treated orally with pinocembrin 10 mg/kg (suspended in CMC 0.5%).

Daily pinocembrin administration started once the rats were already diabetic and for forty days. At the 0, 20 and 40 days the rats were placed into metabolic cages for 24 h. Pinocembrin dose used in the present study was chosen according to literature [26].

II. Corrective scheme

(a) Control (*n* = 6); (b) Pinocembrin (*n* = 6); (c) Diabetic group (*n* = 9); (d) Diabetic + Pinocembrin (*n* = 9) all the groups were treated as above.

Pinocembrin was administrated for twenty days after forty days of untreated hyperglycemia. At the time 0, 20, 40, 50 and 60 days the rats were placed into metabolic cages for 24 h.

At the end of the experiments, the animals were anesthetized with a mixture of isoflurane/oxygen (3%). Blood, urine and kidney were collected. One kidney was frozen in liquid nitrogen and kept at −80 °C until use. The other kidney was perfused with phosphate buffer and 10% formaldehyde and glutaraldehyde for histological studies.

The pinocembrin used in this assay was the pool of pinocembrin isolated from the three samples of propolis.

### 4.8. Biochemical Analyses

Glucose, creatinine, blood urea nitrogen (BUN), total cholesterol, triglycerides, low density lipoprotein (LDL), very low density lipoprotein (VLDL), high density lipoprotein (HDL), alanine aminotransferase (ALT) and aspartate aminotransferase (AST) were quantified in serum. Creatinine was quantified in urine samples. The quantifications were performed in Beckman Coulter laboratory analyzer AU680 Chemistry System. The concentration of protein was measured in tissue homogenates and in urine, employing the bicinchoninic acid assay [50]. Bovine serum albumin was used as standard.

### 4.9. Biomarkers of Renal Damage

Kidney injury molecule-1 (Kim-1), neutrophil gelatinase-associated lipocalin (NGAL) and *N*-acetyl-β-d-glucosaminidase (NAG) were measured by immunoassays (Cloud-Clone Corporation, TX, USA).

### 4.10. Oxidative Stress Markers

The lipid peroxidation was determined through the content of malondialdehyde (MDA) in the kidney according to the methodology previously reported [51]. Tetramethoxypropane was used as standard.

Content of H_2_O_2_ in urine was measured with the commercial kit Amplex Red Hydrogen Peroxide/Peroxidase Assay kit (Molecular Probes, Eugene, OR, USA).

### 4.11. Histopathological Analysis

The kidneys were kept in formaldehyde and embedded in paraffin. Sections of 3 μm were stained with periodic acid-Schiff (PAS). 

Sections of kidney in glutaraldehyde were embedded in Epon resin and contrasted with Reynold’s lead. Ultrafine sections (70 nm) were observed under transmission electron microscope (FEI Company, Hillsboro, OR, USA) and photographs were taken in order to measure glomerular basement membrane (GBM) thickness with a Transmission Electron Microscopy Imaging and Analysis digitizer, version 4.7 SP3 (FEI Company, Hillsboro, OR, USA). The width of GBM was estimated as the perpendicular distance from endothelial cell boundary to the epithelial cell boundary of the peripheral basement membrane [52].

### 4.12. Statistical Analysis

Data were analyzed by one-way ANOVA and Newman Keuls Test for multiple comparisons. Overall survival was calculated with Kaplan Meier analysis. All values are expressed as the mean ± standard error of the mean (SEM). The statistical analyses were performed using GraphPad Prism v.4 software (San Diego, CA, USA) and SPSS v.21.0.0 (Chicago, IL, USA). *p* values < 0.05 were considered significant.

## 5. Conclusions

Flavonoids are the main compounds in northern Mexican propolis and pinocembrin is the major compound.

Pinocembrin had opposite effects depending on the stage of DN. At an early stage, pinocembrin could prevent the progression of damage mainly in the kidneys and dyslipidemias. But at advanced stages, pinocembrin accelerates the progression of the disease. In both effects, oxidative stress plays an important role. 

## Figures and Tables

**Table 1:** Antioxidant Capacity of EEP Samples.

EEP Sample	Total Phenolics mg eq. GA/g Extract	Total Flavonoids mg eq. Q/g Extract	DPPH TE/g Extract	FRAP TE/g Extract	β-Carotene Bleaching Assay %AA
Durango	139 ± 1	90 ± 2	1145 ± 10	20 ± 1	46 ± 13
Zacatecas	109 ± 2	70 ± 1	1098 ± 22	13 ± 1	49 ± 5
Chihuahua	126 ± 3	71 ± 2	975 ± 33	21 ± 1	39 ± 8

**Table 2:** Oxidative Stress Parameters in Preventive Model.

	Control	Pinocembrin	Diabetic	Diabetic + Pinocembrin
MDA Plasma (μM)	0.93 ± 0.19	1.0 ± 0.14	2.2 ± 0.3 ^a^	1.3 ± 0.2 ^b^
MDA Kidney (nM/mg Protein)	21.8 ± 3.7	19.6 ± 2.2	42.2 ± 4.9 ^a^	27.6 ± 2.9 ^b^
Urinary H_2_O_2_ (μM/24h)	0.04 ± 0.04	0.14 ± 0.09	2.7 ± 0.7 ^a^	2.6 ± 0.7 ^a^

Values are Expressed as Means ± SEM. ^a^ *p* < 0.01 vs. Control; ^b^ *p* < 0.01 vs. Diabetic, *n* = 7–8.

**Table 3:** Oxidative stress parameters in corrective model.

	Control	Pinocembrin	Diabetic	Diabetic + Pinocembrin
MDA Plasma	0.92 ± 0.25	1.2 ± 0.3	2.4 ± 0.4 ^a^	1.9 ± 0.2 ^a^
MDA Kidney	18.8 ± 3.1	39.1 ± 2.7^a^	34.2 ± 2.74 ^a^	22.4 ± 3.3 ^b^
Urinary H_2_O_2_ (μM/24h)	0.09 ± 0.04	0.03 ± 0.03	3.7 ± 0.8 ^a^	5.9 ± 1.3 ^a,b^

Values are Expressed as Means ± SEM. ^a^ *p* < 0.01 vs. Control, ^b^ *p* < 0.0001 vs. Diabetic, *n* = 6–8.

## Supplementary Materials

Supplementary materials are available online. Figure S1: ^1^H-NMR of pinocembrin (1), Figure S2: Flavonoids isolated from Mexican propolis. Table S1: ^1^H-RMN data of the flavonoids isolated from Mexican propolis.
