# Supplementary material for: Effect of Pinocembrin Isolated from Mexican Brown Propolis on Diabetic Nephropathy
Source: Molecules. 2018 Apr 9;23(4):852. doi: 10.3390/molecules23040852 (PMC6017349; doi:10.3390/molecules23040852)
Supplement: Supplementary file 1 [file molecules-23-00852-s001.pdf]

molecules

MDPI

Effect of pinocembrin isolated from Mexican brown propolis on diabetic nephropathy

Jessica Granados-Pineda [1,†], Norma Uribe-Uribe [2], Patricia García-López [3], María del Pilar Ramos-Godinez [4], J. Fausto Rivero-Cruz [1,*], Jazmín Marlen Pérez-Rojas [c,*]

[1]Facultad de Química, Universidad Nacional Autónoma de México, Ciudad Universitaria, 04510, CD. MX., México. jesssygpin@hotmail.com (J. G-P.); joserc@unam.mx (J. F. R-C.)
[2]Instituto Nacional de Ciencias Médicas y Nutrición Salvador Zubirán S.S. 14080, CD. MX., México. nofeliauribe@yahoo.com.mx (N. U-U.)
[3]Subdirección de Investigación Básica, Instituto Nacional de Cancerología. 14080, CD. MX., México pgracia_lopez@yahoo.com.mx (P. G-L.); jazminmarlen@gmail.com (J. P-R.)
[4]Departamento de Patología Quirúrgica, Instituto Nacional de Cancerología. 14080, CD. MX., México pilyrg@gmail.com (M. P. R-G.)
[†]Taken in part from the PhD thesis of J. Granados-Pineda
*Correspondence: jazminmarlen@gmail.com; Tel.: Tel.: +52 55 5628 0400. Ext. 32085; joserc@unam.mx; Tel.: +52 55 5622 5281. Fax +52 55 5622 5283.

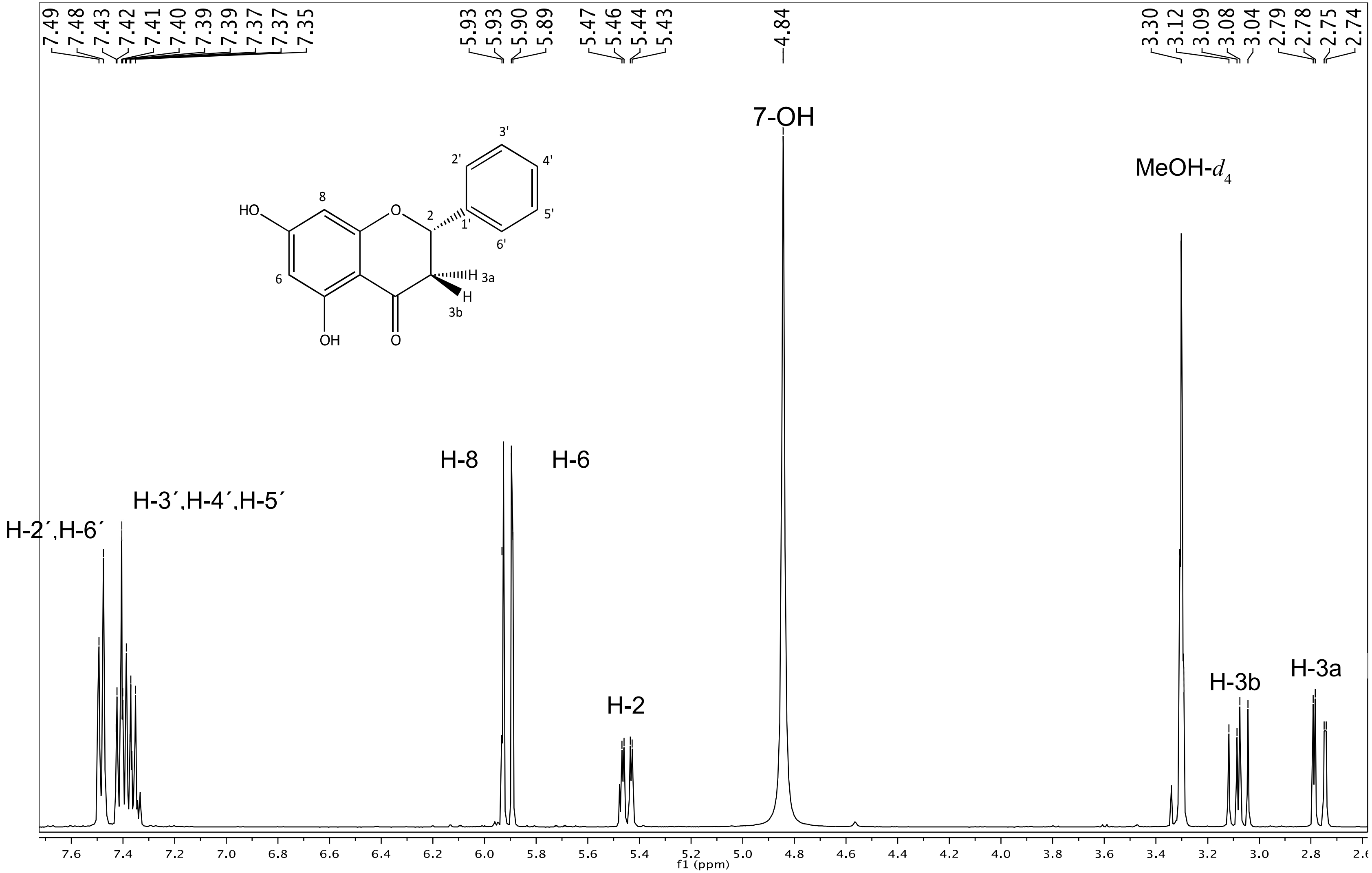

Figure S1. ^{1}H-NMR of pinocembrin (**1**)

molecules

MDPI

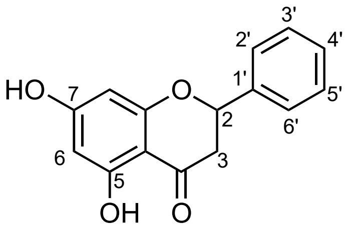

Pinocembrin
(**1**)

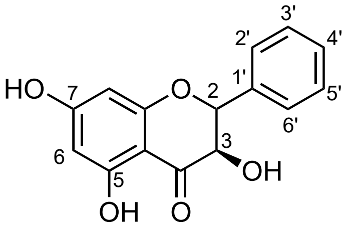

Pinobanksin
(**2**)

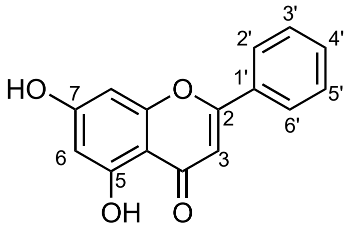

Chrysin
(**3**)

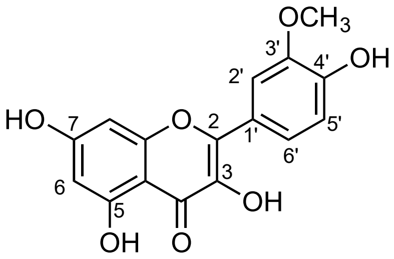

Isorhamnetin
(**4**)

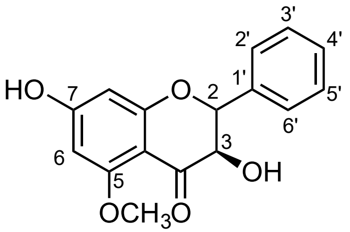

Pinobanksin 5-methylether
(**5**)

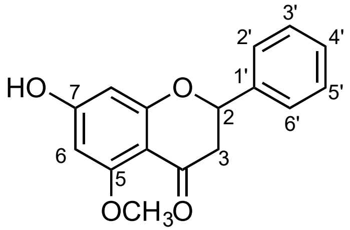

Alpinetin
(**6**)

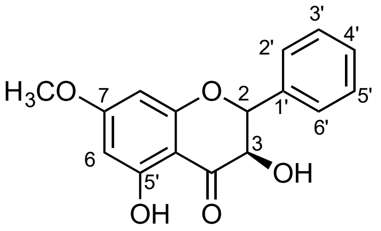

Alpinone
(**7**)

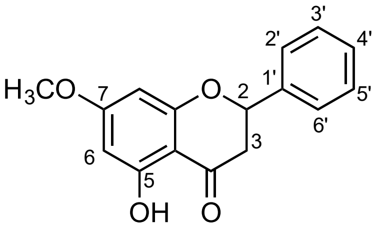

Pinostrobin
(**8**)

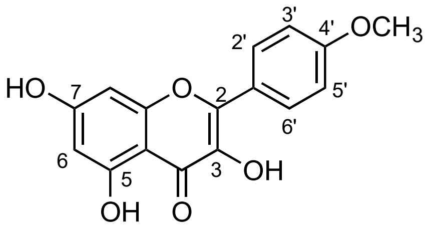

Kaempferide
(**9**)

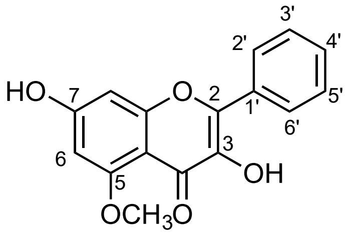

Galangin 5-methylether
(**10**)

Figure S2. Flavonoids isolated from Mexican propolis.

molecules

MDPI

Table S1. ^{1}H NMR data of the flavonoids isolated from Mexican propolis.

H	Pinocembrin (**1**) (MeOH-d_4,) [a]	Pinobanksin (**2**) (acetone-d_6) [a]	Chrysin (**3**) (DMSO-d_6) [a]	Isorhamnetin (**4**) (DMSO-d_6) [a]	Pinobanksin 5-methyether (**5**) (DMSO-d_6)	Alpinetin (**6**) (DMSO-d_6) [a]	Alpinone (**7**) (DMSO-d_6) [a]	Pinostrobin (**8**) (DMSO-d_6) [a]	Kaempferide (**9**) (DMSO-d_6) [a]	Galangin 5-methylether (**10**) (DMSO-d_6) [a]
2	5.45 (1H, dd, *J*=16.0, 4.0)	4.68 (1H, d, *J*=12.0 Hz)	-	-	4.32 (1H, d, *J*=12.0 Hz)	5.46 (1H, dd, *J*=16.0, 4.0 Hz)	5.05 (1H, d, *J*=12.0 Hz)	5.50 (1H, dd, J=10.0, 3.3 Hz)	-	-
3	3a 2.76 (1H, dd, *J*=4.0, 16.0 Hz) 3b 3.09 (1H, dd, *J*=16.0, 12.0 Hz)	5.18 (1H, d, *J*=12.0 Hz)	6.98 (1H, s)	-	5.28 (1H, d, *J*=12.0 Hz)	3a 2.60 (1H, dd, *J*=4.0, 16.0 Hz) 3b 2.97 (1H, dd, J=16.0, 12.0 Hz)	4.40 (1H, d, *J*=12.0 Hz)	3a 2.56 (1H, dd, *J*=13.0, 3.0 Hz) 3b 3.00 (1H, dd, J=13.0, 10.0 Hz)	-	-
6	5.93 (1H, d, *J*=4.0 Hz)	5.98 (1H, s, *J*=2.0 Hz)	6.20 (1H, d, *J*=2.0 Hz)	6.17 (1H, d, *J*=2.0 Hz)	5.94 (1H, d, *J*=2.0 Hz)	5.93 (1H, d, *J*=2.0 Hz)	5.98 (1H, d, *J*=2.0 Hz)	6.00 (1H, d, *J*=2.0 Hz)	6.27 (1H, d, *J*=2.0 Hz)	6.31 (1H, d, *J*=2.0 Hz)
7	-	-	-	-	-	-	-	-	-	-
8	5.90 (1H, d, *J*=4.0 Hz)	6.01 (1H, s, *J*=2.0 Hz)	6.52 (1H, d, *J*=2.0 Hz)	6.45 (1H, d, *J*=2.0 Hz)	6.05 (1H, d, *J*=2.0 Hz)	5.98 (1H, d, *J*=2.0 Hz)	6.10 (1H, d, *J*=2.0 Hz)	6.19 (1H, d, *J*=2.0 Hz)	6.55 (1H, d, *J*=2.0 Hz)	6.50 (1H, d, *J*=2.0 Hz)
2’, 6’	7.49 (2H, dd, *J*=8.0, 4.0 Hz)	7.59 (2H, dd, *J*=8.0, 4.0 Hz)	8.1 (2H, dd, *J*=8.0, 4.0 Hz)	-	7.48 (2H, dd, *J*=8.0, 4.0 Hz)	7.48 (2H, dd, *J*=8.0, 4.0 Hz)	7.51 (2H, dd, *J*=8.0, 4.0 Hz)	7.51 (2H, dd, *J*=8.0, 4.0 Hz)	8.22 (2H, dd, *J*=8.0, 4.0 Hz)	8.12 (2H, dd, *J*=8.0, 4.0 Hz)
3’-5’	7.39 (3H, m)	7.45 (3H, m)	7.55 (3H, m)	-	7.41 (3H, m)	7.41 (3H, m)	7.48 (3H, m)	7.48 (3H, m)	7.13 (3H, m)	7.48 (3H, m)
2´	-	-	-	7.73 (1H, d, *J*=2.0 Hz)	-	-	-	-	-	-
5´	-	-	-	6.92 (1H, d, *J*= 8.0 Hz)	-	-	-	-	-	-
5-OH	-	-	12.63 (1H, s)	12.70 (1H, s)	-	-	10.05 (1H, s)	-	-	-
5-OCH$_3$	-	-	-		3.75 (3H, s)	3.72 (3H, s)	-	-	-	3.57 (3H, s)
7-OCH$_3$	-	-	-	-	-	-	3.82 (3H, s)	3.74 (3H, s)	-	8.93 (1H, s)
3´-OCH$_3$	-	-	-	3.82 (3H, s)	-	-	-	-	-	-
4´-OCH$_3$	-	-	-		-	-	-	-	3.90 (3H, s)	-
4´-OH	-	-	-	9.32 (1H)	-	-	-	-	-	-

[a]400 MHz
